# Aldosterone induces clonal *β*-cell failure through glucocorticoid receptor

**DOI:** 10.1038/srep13215

**Published:** 2015-08-19

**Authors:** Fang Chen, Jia Liu, Yanyang Wang, Tijun Wu, Wei Shan, Yunxia Zhu, Xiao Han

**Affiliations:** 1 Key Laboratory of Human Functional Genomics of Jiangsu Province, Nanjing Medical University, 140 Hanzhong Road, Nanjing 210029, China

## Abstract

Aldosterone excess causes insulin resistance in peripheral tissues and directly impairs the function of clonal *β*-cell. The aim of this study was to investigate the molecular mechanisms involved in the aldosterone-induced impairment of clonal *β*-cells. As expected, aldosterone induced apoptosis and *β*-cell dysfunction, including impairment of insulin synthesis and secretion, which were reversed by Glucocorticoid receptor (GR) antagonists or GR-specific siRNA. However, mineralocorticoid receptor (MR) antagonists or MR-specific siRNA had no effect on impairment of clonal *β*-cells induced by aldosterone. Besides, aldosterone significantly decreased expression and activity of MafA, while activated JNK and p38 MAPK in a GR-dependent manner. In addition, JNK inhibitors (SP600125) and/or p38 inhibitors (SB203580) could abolish the effect of aldosterone on MafA expression and activity. Importantly, overexpression of JNK1 or p38 reversed the protective effect of a GR antagonist on the decrease of MafA expression and activity. Furthermore, aldosterone inhibits MafA expression at the transcriptional and post-transcriptional level through activation of JNK and p38, respectively. Consequently, overexpression of MafA increased synthesis and secretion of insulin, and decreased apoptosis in clonal *β*-cells exposed to aldosterone. These findings identified aldosterone as an inducer of clonal *β*-cell failure that operates through the GR-MAPK-MafA signaling pathway.

Aldosterone is a key component of the renin–angiotensin–aldosterone system (RAAS) and plays a pivotal role in sodium reabsorption and potassium secretion^1^. However, aldosterone is also associated with the pathogenesis of metabolic syndrome if it is inappropriately elevated in obese individuals^2^. This correlation has led investigators to explore the role of aldosterone in the development of diabetes mellitus. Plasma aldosterone concentration is reported to increase significantly in patients with diabetes mellitus when compared to healthy controls^3^,^4^. Aldosterone excess induces insulin resistance in peripheral tissues and it also directly impairs pancreatic *β*-cell^5^,^6^. In recent years, most studies have focused on the mechanism of underlying the aldosterone induction of insulin resistance, while little research has been conducted to investigate how aldosterone initiates pancreagtic *β*-cell failure.

The classic aldosterone signaling pathway depends on the mineralocorticoid receptor (MR) in various tissues such as kidney, lung and vascular endothelial cells^7^,^8^. In pancreatic *β*-cells, the expression of MR protein is low and aldosterone exerts its actions in an MR-independent manner^6^,^9^. The involvement of other receptors has been confirmed for the effect of aldosterone^10^: for example, aldosterone regulates target gene expression by binding to the glucocorticoid receptor (GR) in adipocytes^11^. Clonal *β*-cells show a much more abundant expression level of GR than of MR. In addition, increased GR activity is sufficient for development of the diabetic state, while knock-down GR expression blocks the reduction of *β*-cell mass caused by high dietary intake^12^,^13^. These findings suggest that the induction of *β*-cells failure by aldosterone is mediated by GR.

Pancreatic *β*-cell function and *β*-cell mass play a critical role in the control of blood glucose concentration within a narrow range^14^. The transcription factor MafA (v-maf musculoaponeurotic fibrosarcoma oncogene homologue A) is an activator of insulin synthesis and secretion as well as a key regulator of *β*-cell survival^15^,^16^. Under diabetic conditions, expression and/or transcriptional activity of MafA are decreased, which results in *β*-cells apoptosis and dysfunction^17^. Several factors, such as high levels of glucose, palmitate, and proinflammatory cytokines, inhibited MafA expression in *β*-cells^18^,^19^. In recent years, a growing body of evidence indicated that activation of MAPKs signaling is involved in the regulation of MafA expression^20^,^21^. The MAPKs, including c-Jun N-terminal kinase (JNK), ERK, and p38, are important mediators in the development of diabetes^22^. In the case of MafA, JNK and p38 regulated MafA expression at the transcriptional and post-transcriptional levels, respectively^23^,^24^.

The current study was designed to examine the effects of aldosterone on *β*-cell function and mass. We found that aldosterone stimulated GR expression and increased JNK and p38 phosphorylation level while decreasing MafA level and activity. We therefore tested the hypothesis that aldosterone directly induces *β*-cell dysfunction and apoptosis by promoting the GR regulation of MafA expression via MAPK activation.

## Results

### Aldosterone induced dysfunction and apoptosis of clonal *β*-cell

**Figure 1:**
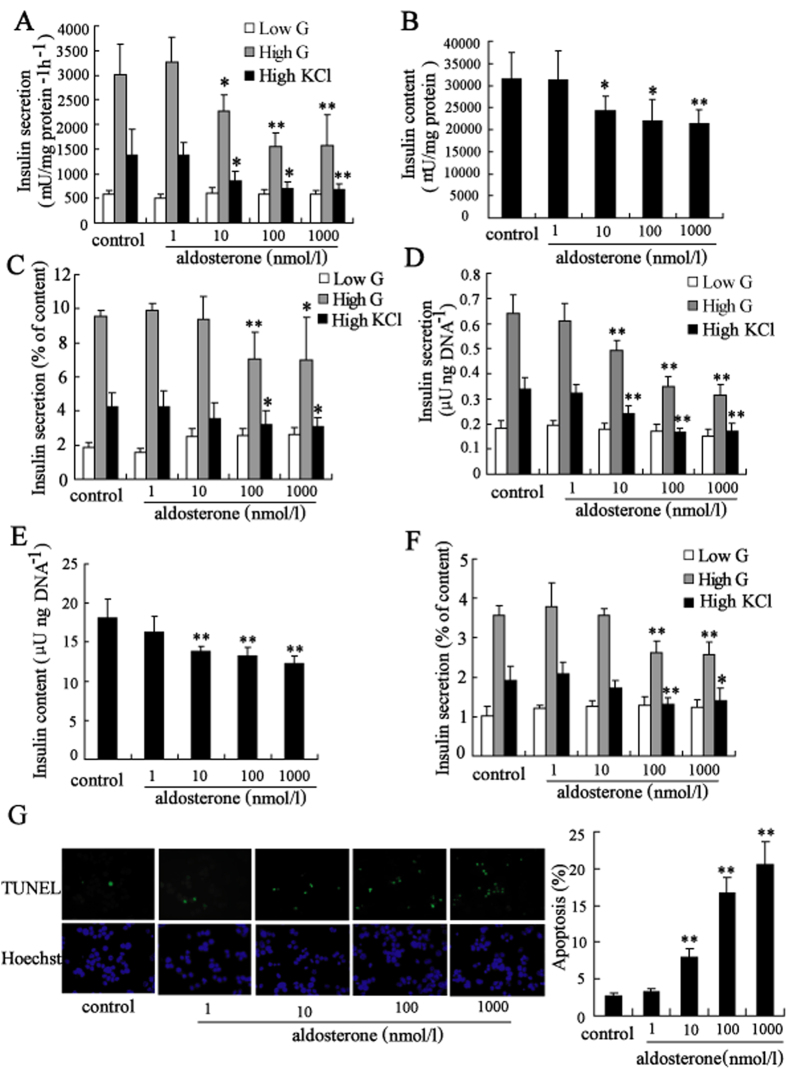
Aldosterone induced dysfunction and apoptosis of clonal *β*-cell. Treatment with aldosterone (10, 100, 1000 nmol/l) for 24 h significantly decreased (**A**) insulin secretion (white bars, 2 mmol/l glucose; gray bars, 20 mmol/l glucose; black bars, 50 mmol/l KCl), (**B**) insulin content and (**C**) ratio of secreted insulin to insulin content represented as percentage of Min6 cells. Similar effects on (**D**) insulin secretion (white bars, 2 mmol/l glucose; gray bars, 20 mmol/l glucose; black bars, 50 mmol/l KCl), (**E**) insulin content of mouse islets and (**F**) ratio of secreted insulin to insulin content represented as percentage of mouse islets were observed. (**G**) Treatment with aldosterone (10, 100, 1000 nmol/l) for 72 h significantly induced apoptosis of Min6 cells measured by staining with TUNEL and Hoechst. Apoptosis was determined by scoring the percentage of TUNEL-positive cells. About 2,000 cells were scored for each group in one experiment. *P < 0.05 and **P < 0.01, compared to control.

Aldosterone significantly decreased insulin secretion from Min6 cells after stimulation with 20 mmol/l glucose or 50 mmol/l KCl in a dose-dependent manner when compared to the non-treated control. As shown in Fig. 1A, insulin secretion in Min6 cells after stimulation with high glucose was decreased to 75.5%, 51.4%, and 52.3% after treatment with 10 nmol/l, 100 nmol/l, and 1000 nmol/l aldosterone for 24 hours, respectively. Besides, insulin secretion in Min6 cells after stimulation with high KCl was decreased to 61.9%, 50.8%, and 49.6% after treatment with 10 nmol/l, 100 nmol/l, and 1000 nmol/l aldosterone for 24 hours, respectively. Furthermore, insulin content was decreased in Min6 cells after treatment with aldosterone (Fig. 1B). After adjustment for insulin content, high glucose-stimulated and high KCl-stimulated insulin secretion was also significantly decreased in Min6 cells after treatment with 100 nmol/l, and 1000 nmol/l aldosterone (Fig. 1C). Similar results were observed in mouse islets after treatment with aldosterone for 24 h (Fig. 1D–F). The INS-1 cells did not respond to glucose, so we performed the potassium-stimulated-insulin secretion (KSIS assay) to evaluate insulin secretion ability. The effect of aldosterone treatment on insulin secretory function and synthesis in INS-1 cells and rat islets was consistent with that in Min6 cells and mouse islets (supplemental Fig. 1A–D).

*β*-cell viability measured using MTT assays revealed that aldosterone treatment significantly reduced the viability of Min6 and INS-1 cells in a time- and dose-dependent manner (supplemental Fig. 1E,F). Programmed islet cell death evaluated by scoring the percentage of TUNEL-positive cells (Fig. 1G and supplemental Fig. 1G), indicated a significant increase in apoptosis in cells exposed to 100 nmol/l aldosterone for 72 h.

### Aldosterone induced impairment of clonal *β*-cells in a GR-dependent manner

**Figure 2:**
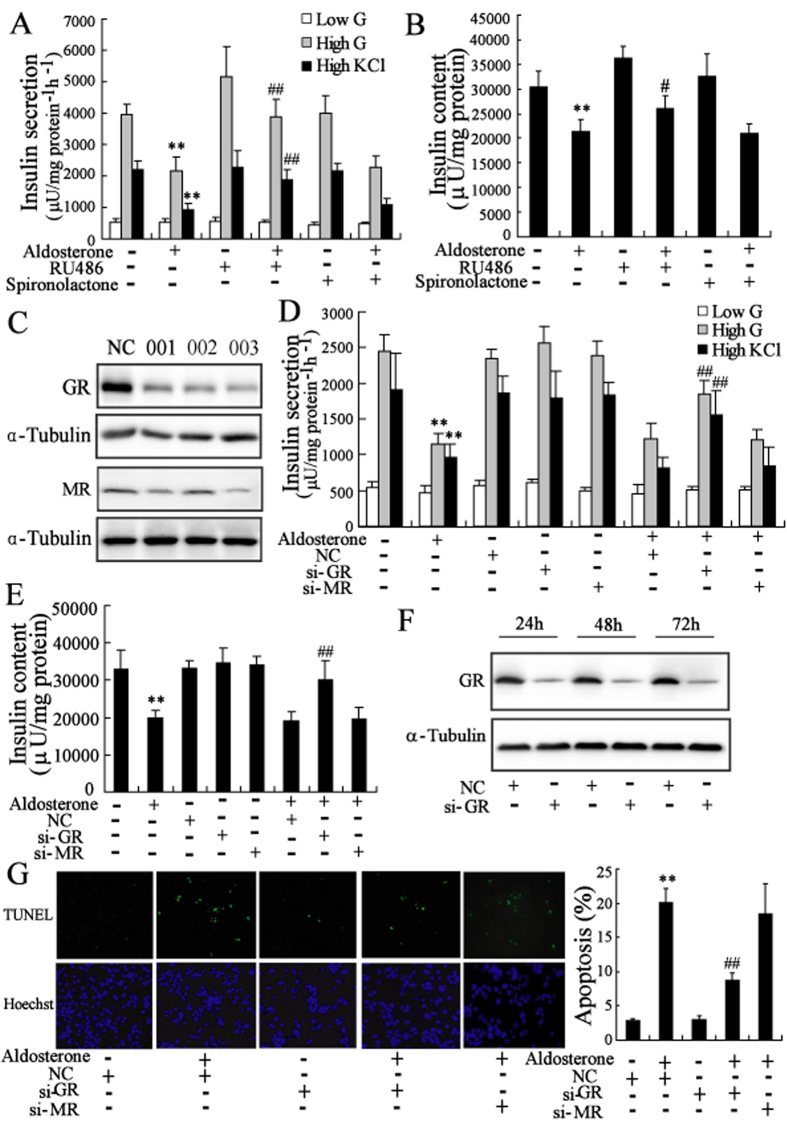
Aldosterone induced impairement of clonal *β*-cells in a GR-dependent manner. After pretreatment with MR antagonist spironolactone (100 nmol/l) or GR antagonist RU486 (1 μmol/l) for 2 h, Min6 cells were treated with the aldosterone for additional 24 h. The decrease of insulin secretion (**A**) and insulin content (**B**) in Min6 cells induced by aldosterone were significantly reversed by RU486 pretreatment. (**C**) Transfected with 100 nmol/l GR siRNAs (001, 002, 003) in Min6 cells for 24 h significantly downregulated GR protein expression. Similar results were obtained by transfected with MR siRNAs (001, 002, 003). After transfected with 100 nmol/l NC, si-GR (003) or si-MR (003) for 24 h, Min6 cells were treated with aldosterone (100 nmol/l) for 24 h. The decrease of insulin secretion (**D**) and insulin content (**E**) in Min6 cells induced by aldosterone were significantly reversed by transfected with si-GR. (**F**) Transfected with 100 nmol/l si-GR (003) significantly downregulated GR protein expression in Min6 cells for different time. (**G**) Min6 cells were transfected with 100 nmol/l NC, si-GR (003) or si-MR (003) for 24 h, and then treated with aldosterone (100 nmol/l) for 72 h. The apoptosis of Min6 cells induced by aldosterone were significantly reversed by transfected with si-GR. **P < 0.01, compared to control; ##*P* < 0.01, compared to NC + aldosterone group.

MR antagonist spironolactone (100 nmol/l) did not prevent the deterioration of *β*-cells induced by aldosterone, whereas pretreatment with GR antagonist RU486 (1 μmol/l) prevented the effects of aldosterone on insulin secretion and synthesis (Fig. 2A,B and supplemental Fig. 2A,B).

We next used MR-specific siRNA (003) and GR-specific siRNA (003) to induce clear decreases in the MR and GR expression, respectively (Fig. 2C,F and supplemental Fig. 2C,F). We further identified the effects of reduced MR expression and GR expression in Min6 cells treated with aldosterone. The results showed that knock-down of GR expression prevented the inhibition of insulin secretion and synthesis as well as *β*-cell apoptosis induced by aldosterone (Fig. 2D,E,G). However, knock-down of MR expression had no effect on clonal *β*-cell failure induced by aldosterone. Similar results were observed in INS-1 cells (supplemental Fig. 2D,E,G).

### Aldosterone treatment results in a decrease in MafA expression level and transcriptional activity in GR-dependent pathway

**Figure 3:**
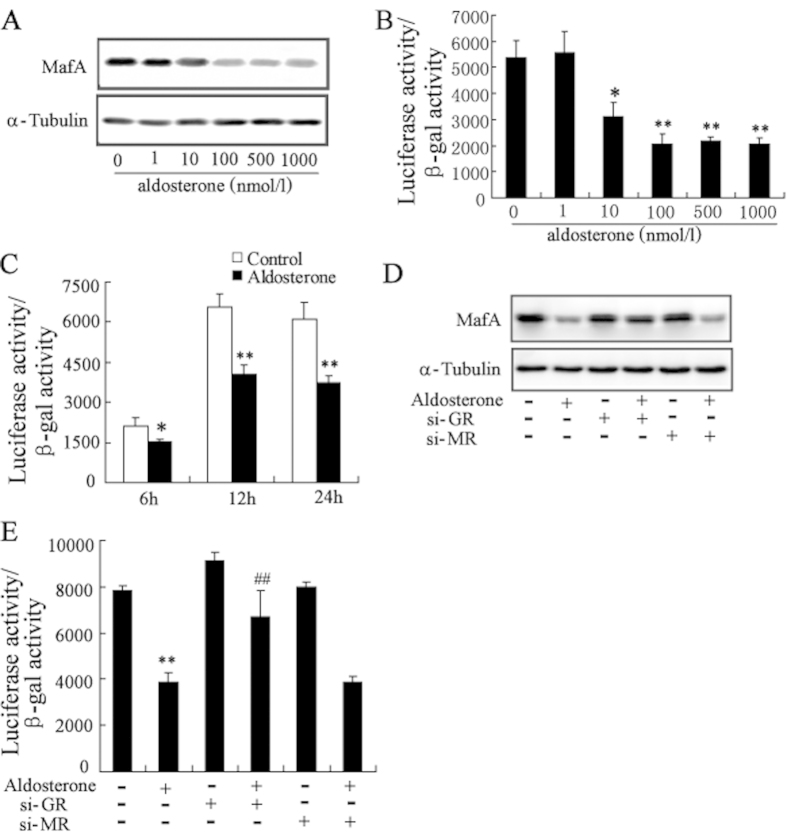
Aldosterone treatment results in a decrease in MafA protein level and transcriptional activity in GR-dependent pathway. (**A**) Treatment with aldosterone in Min6 cells for 24 h significantly decreased the protein levels of MafA in a dose-dependent manner. (**B**) Min6 cells were transfected with MAREs-luc reporter plasmid for 24 h, then, treated with aldosterone for 24 h. Treatment with aldosterone significantly decreased MafA transcriptional activity in a dose-dependent manner. (**C**) Min6 cells were transfected with MAREs-luc reporter plasmid for 24 h, then, treated with aldosterone for 6 h, 12 h and 24 h. Treatment with aldosterone significantly decreased MafA transcriptional activity in a time-dependent manner. After transfection with si-GR or si-MR for 24 h, Min6 cells were treated with aldosterone (100 nmol/l) for 24 h. The decrease of MafA protein level (**D**) and MafA transcriptional activity (**E**) in Min6 cells induced by aldosterone was significantly reversed by transfected with si-GR. *P < 0.05 and **P < 0.01, compared to control, ##*P* < 0.01, compared to aldosterone-treated group.

In Min6 cells, a large and dose-dependent decrease in MafA protein levels was observed following treatment with aldosterone for 24h (Fig. 3A). We also examined the relationship between aldosterone treatment and MafA transcriptional activity by transfecting Min6 cells with the MAREs-luc reporter plasmid where luciferase expression is controlled by an insulin promoter containing MafA binding sites^25^. The luciferase activity was decreased in a dose- and time-dependent manner in aldosterone-treated cells when compared with controls (Fig. 3B,C).

Treatment with aldosterone also dramatically decreased MafA protein levels, and this effect could be reversed by inhibition of GR expression (Fig. 3D). In addition, treatment with GR siRNA restored MafA transcriptional activity inhibited by aldosterone (Fig. 3E). However, inhibition of MR expression had no effect on MafA protein levels and activity in aldosterone-treated *β*-cells.

### Activation of MAPK signaling pathway results in the decrease of MafA expression level and transcriptional activity by the treatment of aldosterone

**Figure 4:**
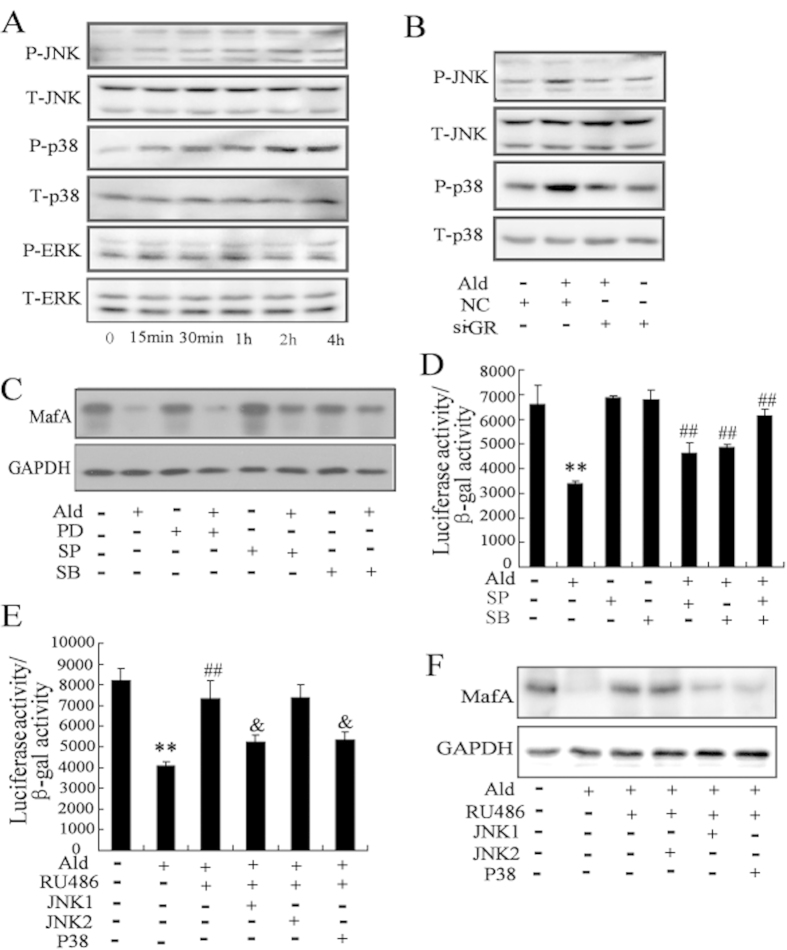
Activation of MAPK signaling pathway results in the decrease of MafA expression level and transcriptional activity by the treatment of aldosterone. (**A**) Treated with aldosterone (100 nmol/l) significantly increased the phosphorylation levels of JNK and p38 in Min6 cells. (**B**) Min6 cells were transfected with si-GR for 24 h, and then treated with aldosterone. The phosphorylation levels of JNK and p38 in Min6 cells induced by aldosterone were significantly reversed by transfected with si-GR. After pretreatment with SP (the JNK-specific inhibitor), SB (the p38-specific inhibitor), and PD (the ERK-specific inhibitor) for 30 min, Min6 cells were treated with aldosterone. The decrease of MafA protein level (**C**) and MafA transcriptional activity (**D**) in Min6 cells induced by aldosterone were significantly reversed by SP and SB. (**E**) Min6 cells were cotransfected with JNK1, JNK2, or P38 expressing plasmids with the MAREs-luc reporter plasmid, and then treated with aldosterone and/or RU486. RU486 attenuated the inhibitory effects of aldosterone on MafA transcriptional activity, which was reversed by overexpression of JNK1 or p38. (**F**) Min6 cells were transfected with JNK1, JNK2, or P38 expressing plasmids, and then treated with aldosterone and/or RU486. RU486 attenuated the inhibitory effects of aldosterone on MafA protein level, which was reversed by overexpression of JNK1 or p38. **P < 0.01, compared to control; ##*P* < 0.01, compared to aldosterone treatment. & *P* < 0.01, compared to aldosterone + RU486.

As shown in Fig. 4A, aldosterone increased the phosphorylation levels of JNK and p38 in a time-dependent manner in Min6 cells. However, aldosterone treatment had no effect on the phosphorylation level of ERK. Inhibition of GR expression suppressed aldosterone-mediated activation of JNK and p38 (Fig. 4B), indicating that aldosterone-induced JNK and p38 MAPK activation in *β*-cells involves a GR-dependent pathway.

A 30-min pretreatment with the JNK-specific inhibitor SP600125, or the p38-specific inhibitor SB203580, followed by incubation with aldosterone for 12 h resulted in a partial reversal of the reduction in the MafA protein levels due to aldosterone (Fig. 4C). By contrast, the ERK-specific inhibitor PD98059 had no obvious effect on MafA expression changes due to aldosterone-treatment. Consistent with these observations, MafA transcriptional activity was clearly decreased by aldosterone treatment, but could be restored by SP600125 or SB203580, and especially by a combination of SP600125 and SB203580 (Fig. 4D). We further examined the role of MAPK in the GR-mediated decrease of MafA expression and transcriptional activity by cotransfecting Min6 cells with JNK1, JNK2 or p38 plasmids in combination with the MAREs-luc reporter plasmid followed by treatment with aldosterone and/or GR antagonist RU486. RU486 attenuated the inhibitory effects of aldosterone on MafA expression and transcriptional activity. The JNK1 or p38 plasmids reversed the attenuation effect of RU486 (Fig. 4E).

### Aldosterone-induced inhibition of MafA expression is regulated at the transcriptional level by JNK while at post-transcriptional level by P38

**Figure 5:**
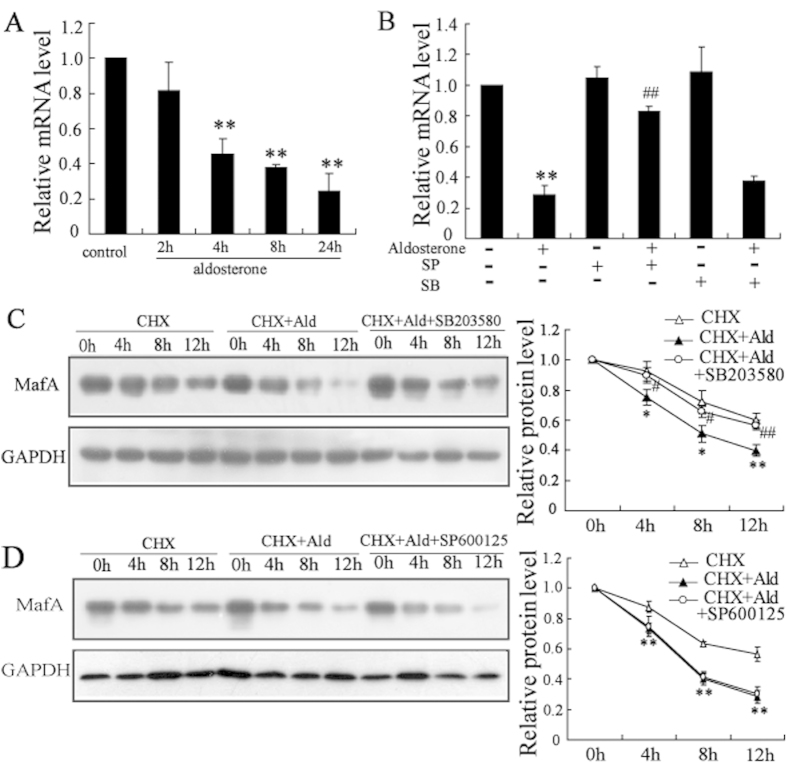
Aldosterone-induced inhibition of MafA expression is regulated at the transcriptional level by JNK while at post-transcriptional level by P38. (**A**) Treatment with aldosterone (100 nmol/l) significantly suppressed MafA mRNA expression in a time-dependent manner in Min6 cells. (**B**) SP (the JNK-specific inhibitor) attenuated the inhibitory effects of aldosterone on MafA mRNA level in Min6 cells. (**C**) Min6 cells were divided into three groups (control, aldosterone (Ald) and Ald + SB203580). After the indicated treatments for 2 h, cells were co-cultured with 50 mmol/l cycloheximide (CHX) for 0, 4, 8, or 12 h. Aldosterone induced a more rapid reduction in MafA protein levels in the presence of CHX, which could be reversed by SB203580. The half-life of MafA was calculated. (**D**) Min6 cells were divided into three groups (control, Ald and Ald + SP600125). SP600125 could not reverse the reduction of MafA protein level induced by aldosterone. The half-life of MafA was calculated. *P < 0.05 and **P < 0.01, compared to control; #*P* < 0.05 and ##P < 0.01, compared to aldosterone treatment.

Aldosterone treatment time-dependently decreased MafA mRNA levels (Fig. 5A), and this decrease could be reversed by SP600125 (the JNK-specific inhibitor) but not by SB203580 (the p38-specific inhibitor) in Min6 cells (Fig. 5B). We further explored whether aldosterone decreased MafA expression at the post-transcriptional level by determining the stability of the MafA protein in Min6 cells treated with aldosterone for 0, 4, 8, or 12 h in the presence of 50 mmol/l cycloheximide (the inhibitor of protein biosynthesis in eukaryotic organisms, CHX). The blockage of *de novo* protein synthesis by CHX resulted in a more rapid reduction in MafA protein levels by aldosterone when compared with control cells. Treatment with SB203580 (Fig. 5C) but not SP600125 (Fig. 5D) stabilized the MafA protein levels.

### Up-regulation of MafA expression protects *β*-cells from aldosterone-induced impairment

**Figure 6:**
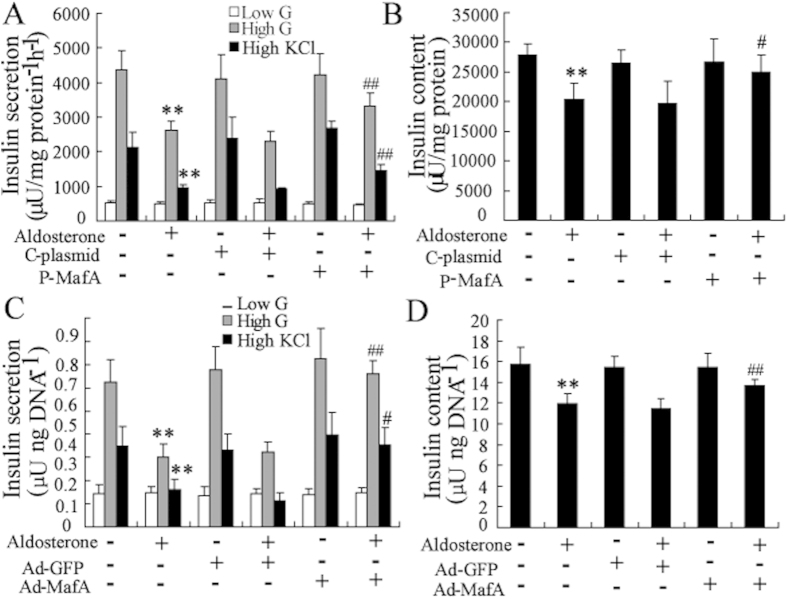
Up-regulation of MafA expression protects *β*-cells from aldosterone-induced the decrease of insulin synthesis and secretion. After transfection with the MafA over-expression plasmid (P-MafA) or control plasmid (C-plasmid) for 24 h, Min6 cells were treated with aldosterone (100 nmol/l) for an additional 24 h. Transfection with P-MafA in Min6 cells reversed the aldosterone-induced decrease of insulin secretion (**A**) and insulin content (**B**). After infection with the MafA over-expression Adenovirus (Ad-MafA) or control Adenovirus (Ad-GFP) for 24 h, mouse islets were treated with aldosterone (100 nmol/l) for an additional 24 h. Infection with Ad-MafA in mouse islets reversed the aldosterone-induced decrease of insulin secretion (**C**) and insulin content (**D**). **P < 0.01, compared to control. #P < 0.05 and ##P < 0.01, compared to C-plasmid or Ad-GFP combined with aldosterone-treated group.

The overexpression potencies of these plasmid and adenoviruses were confirmed in cell lines and primary islets by Western blotting (supplemental Fig. 3A). Overexpression of MafA in Min6 and mouse islets reversed the aldosterone-induced impairment of insulin secretion and synthesis (Fig. 6A–D). Similar results were observed in INS-1 cells and rat islets (supplemental Fig. 3B–E).

**Figure 7:**
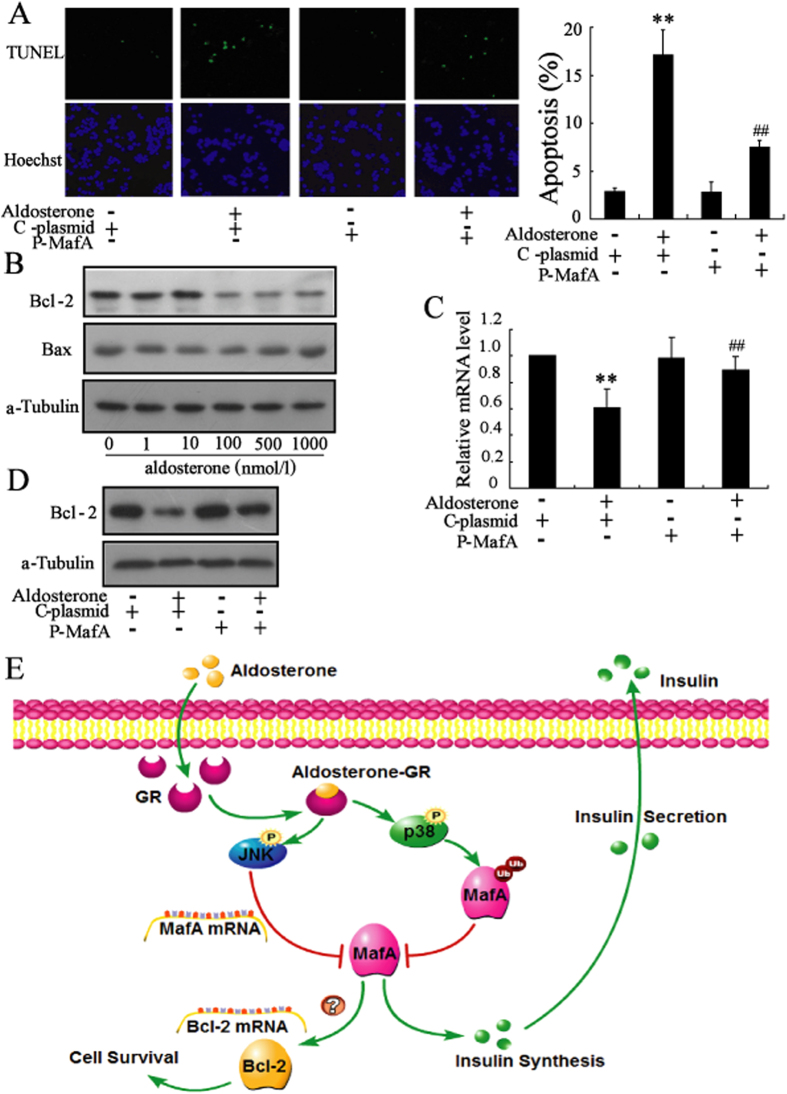
Up-regulation of MafA expression protects *β*-cells from aldosterone-induced apoptosis. (**A**) After transfection with the MafA over-expression plasmid (P-MafA) or control plasmid (C-plasmid) for 24 h, Min6 cells were treated with aldosterone (100 nmol/L) for an additional 72 h. Transfection with P-MafA reversed the aldosterone-induced apoptosis of Min6 cells. (**B**) Aldosterone significantly decreased the protein level of Bcl-2 in a dose-dependent manner. (**C**) Transfection with P-MafA in Min6 cells reversed the aldosterone-induced decrease of Bcl-2 mRNA level. (**D**) Transfection with P-MafA in Min6 cells reversed the aldosterone-induced decrease of Bcl-2 protein level. (**E**) Diagram depicting the mechanism of aldosterone-induced *β*-cell failure. **P < 0.01, compared to control. ##P < 0.01, compared to C-plasmid combined with aldosterone-treated group.

Consistent with the effect of GR-siRNA, overexpression of MafA decreased *β*-cell apoptosis induced by aldosterone (Fig. 7A and supplemental Fig. 3F). We explored the mechanism of MafA protected *β*-cells against aldosterone-induced apoptosis by measuring the expression of Bcl-2 family proteins, including anti-apoptotic Bcl-2 protein and pro-apoptotic Bax protein. We found that aldosterone treatment of Min6 cells induced a significant decrease in Bcl-2 expression but had no effect on Bax expression (Fig. 7B). Overexpression of MafA in Min6 cells and INS-1 cells reversed the aldosterone-induced decrease in Bcl-2 expression (Fig. 7C,D).

## Discussion

Recent studies demonstrate that excess aldosterone induces dysfunction and apoptosis of pancreatic *β*-cells^6^,^9^. However, the molecular mechanism under the aldosterone-mediated impairment of *β*-cells remains poorly understood. In the present study, we found that short-term exposure to aldosterone impaired *β*-cell function and that long-term treatment of aldosterone induced cytotoxicity and apoptosis of *β*-cells. Our data indicate that aldosterone induces impairment of *β*-cells mainly via GR activating MAPK (JNK and p38) so that to reduce the expression and activity of MafA (Fig. 7E).

Previous studies have demonstrated that aldosterone impaired glucose-stimulated insulin secretion but had no effect on insulin content in Min6 cells and mouse islets^6^. However, in the present study, we found that aldosterone decreased insulin secretion and insulin content in Min6 and INS-1 cells, as well as isolated mouse and rat islets. This discrepancy could be partly explained by that Min6 cells displayed functional heterogeneity during cell culture and expressed different gene among different subclones^26^,^27^. Besides, techniques of isolated islets were different between two laboratories, which may contribute to the discrepancy between the results of the islets^28^,^29^. Interestingly, our data showed that insulin secretion adjusted for insulin content was still significantly decreased in Min6 cells and mouse islets treated with aldosterone. These results suggested that both insulin secretory capacity and insulin synthesis were affected by aldosterone.

GR, a ligand-activated transcription factor, is closely associated with fetal *β*-cell development and mature *β*-cell function. It was reported that loss of GR in clonalprecursors, but not in mature *β*-cell cells, increases *β*-cell mass^30^. Transgenic mice with specific overexpression of GR in clonal *β*-cell displayed a reduced glucose tolerance due to *β*-cell failure^31^. Moreover, GR activation inhibited expression of insulin gene in human^32^. In this study, we found that the GR antagonist prevented the impairment of *β*-cells by aldosterone, suggesting the impairment was mediated by GR. However, Luther *et al.* reported that GR antagonist did not prevent the damage effect of aldosterone on insulin secretion in isolated mouse islets^6^. Different levels and durations of GR inactivation and species-specific difference in the response to antagonist may account for the different effects of GR antagonism on aldosterone-mediated impairment of *β*-cells^33^,^34^. We further used siRNA specific for GR to silence endogenous GR expression to determine whether the effects of aldosterone were mediated through GR in *β*-cells. GR-specific siRNA partially abolished aldosterone-mediated impairment in mouse MIN6 cells (Fig. 2D,E,G) and rat INS-1 cells (supplemental Fig. 2D,E,G), as well as isolated mouse and rat islets (data not shown). These findings, taken together, suggest that aldosterone treatment is mediated in part by activation of GR.

In clonal *β*-cells, the expression level of MR is low compared to that of GR (Fig. 2C and Fig. S2C). We found that MR antagonist spironolactone and MR-specific siRNA did not prevent impairment of *β*-cells by aldosterone. This result indicated that aldosterone exerted its damaging effects on *β*-cells via an MR-independent mechanism, consistent with previous reports^6^,^9^.

MafA, a basic leucine zipper transcription factor, is exclusively expressed in the beta-cells of the islet and is involved in insulin gene transcription and insulin secretion^35^. This transcription factor has been proposed to be a master regulator of adult islet beta-cell function^15^. Chronic high glucose, palmitate, and proinflammatory cytokines inhibited MafA expression and activity, which led to *β*-cells failure^18^,^19^. As is known to us, this is the first study to demonstrate that aldosterone induced a decrease of MafA expression and activity in GR-dependent pathway. More importantly, overexpression of MafA reverses aldosterone-induced impairment on insulin function and apoptosis in clonal *β*-cells. Thus, our study provides a novel example of the central role of MafA in aldosterone-mediated failure of clonal *β*-cells.

A growing body of evidence suggests that MAPK activation plays an important role in different cellular responses to aldosterone stimuli^36^,^37^. The MAPKs, including c-Jun N-terminal kinase (JNK), ERK, and p38, are important mediators of *β*-cells failure in the development of diabetes^22^. In this study, we found that aldosterone phosphorylated and activated JNK and P38, but had no significant effect on the activity of ERK. Moreover, using specific inhibitors for JNK or p38, we demonstrated that JNK and p38 had a positive synergistic effect of aldosterone on MafA expression and activity. Our previous study showed that the activation of JNK1 and JNK2, the JNK subgroup, led to different functional consequences^38^. Consistent with that, overexpression of JNK1 or p38, but not JNK2 partially abolished the protective effect of a GR antagonist on the aldosterone-induced decrease of MafA expression and transcriptional activity. Thus, the link between MAPK signaling and suppression of MafA was confirmed to involve a GR-mediated effect of aldosterone on clonal *β*-cells.

Previous studies have demonstrated that JNK and p38 MAPK could regulate MafA expression at the transcriptional and post-transcriptional levels, respectively^23^,^24^. In the current studies, we found that JNK inhibitors abolished the decrease in MafA mRNA levels induced by aldosterone but had no effect on stabilization of MafA protein levels, whereas, p38 inhibitors prevented the reduction in MafA protein levels mediated by aldosterone but had no effect on the decrease in MafA mRNA levels. Therefore, aldosterone inhibits MafA expression at the transcriptional or post-transcriptional level through activation of JNK and p38 in clonal *β*-cells, respectively.

In conclusion, our observations suggest that aldosterone affects *β*-cell function by GR-MAPK-MafA signaling pathways, leading to impaired *β*-cell function and ultimately to apoptosis. This research shed light on the mechanisms of aldosterone-induced clonal *β*-cell failure and provided targets for possible interventions during the development of this disorder.

## Materials and Methods

### Reagents

RPMI-1640, DMEM, Trizol, and Lipofectamine 2000 transfection reagent were purchased from Invitrogen Life Technologies (Grand Island, NY, USA). Cycloheximide (CHX), spironolactone (SPL), RU486, PD98059, SB203580, SP600125, type V collagenase and *α*-tubulin antibody were obtained from Sigma Aldrich (St. Louis, MO). Phospho- and total-JNK, phospho- and total-Erk, and phospho- and total-p38 MAPK antibodies were purchased from Cell Signaling Technology (Beverly, MA, USA). GR, MR, Bax, Bcl-2, MafA antibodies, goat anti-rabbit IgG-HRP, and goat anti-mouse IgG-HRP were from Santa Cruz Biotechnology (Santa Cruz, CA, USA). The Detergent Compatible (DC) Protein Assay kit was purchased from Bio-Rad Laboratories (Hercules, CA, USA). The Luciferase Assay System was obtained from Promega (Madison, WI, USA). The firefly luciferase reporter construct MARE-Luc (containing MafA-binding sites) was a kind gift from Dongming Su (Nanjing Medical University, Nanjing, China). The pcDNA3-JNK2 expression vector was kindly provided by Zhimin Yin (Nanjing Normal University, Nanjing, China).

**Cell culture.** Mouse Min6 and rat INS-1 cell lines were established as described previously^39^,^40^. MIN6 cells were grown in DMEM medium (glucose concentration: 25 mmol/l) containing 15% charcoal-stripped FBS and INS-1 cells were grown in RPMI 1640 medium (glucose concentration: 11.1 mmol/l) supplemented with 10% charcoal-stripped FBS. All cells were cultured at 37 °C in a humidified atmosphere containing 95% air and 5% CO_2_. MIN6 and INS-1 cells were treated with aldosterone (0 nmol/l, 10 nmol/l, 100 nmol/l and 1000 nmol/l) and/or SPL (100 nmol/l) or RU486 (1 μmol/l) for 24 or 72 hours. For compounds prepared in ethanol or DMSO, the final concentration of ethanol or DMSO in the culture medium was kept below 0.1% (vol. /vol.). Vehicle controls were prepared for all treatments.

### Isolation and culture of islet

Male Sprague-Dawley rats (250–300 g) and male ICR mice (20–25 g) were purchased from Nanjing Medical University Laboratory Animal Centre, Nanjing, China. Islets were isolated and cultured using previously described techniques^29^. The islets were allowed to equilibrate for 4 hours and counted, and then re-picked into static incubation tubes and cultured overnight for further studies. Mouse and rat islets were treated with aldosterone (0 nmol/l, 10 nmol/l, 100 nmol/l and 1000 nmol/l) and/or SPL (100 nmol/l) or RU486 (1 μmol/l) for 24 hours. All animal studies were performed according to guidelines established by the Research Animal Care Committee of Nanjing Medical University, China. Animals were treated humanely, using approved procedures in accordance with the guidelines of the Institutional Animal Care and Use Committee at Nanjing Medical University, China.

### Western blot analysis

Clonal *β*-cells and isolated primary islets were lysed with ice-cold lysis buffer (50 mmol/l Tris–HCl, pH 7.4; 1% NP-40; 150 mmol/l NaCl; 1 mmol/l EDTA; 1 mmol/l phenylmethylsulfonyl fluoride). Protein concentration in the cell lysate was determined using the DC protein assay kit (Bio-Rad) and the proteins were analyzed by western blotting as previously described^41^.

### Real-time PCR assay

The total RNA of Min6 and INS-1 cells were extracted in TRIzol reagent (Invitrogen, Grand Island, NY, USA) according to the manufacturer’s protocol. After spectrophotometry quantification, 1 μg total RNA was used for reverse transcription in a 20 μl final volume with AMV Reverse Transcriptase (Promega, Madison, WI, USA) according to the manufacturer’s instructions. Quantification of mRNA by real-time PCR was performed using a LightCycler480 II Sequence Detection System (Roche, Basel, Switzerland). The reaction system (20 μl) contained the corresponding cDNA, forward and reverse primers, and SYBR GREEN PCR Master Mix (Applied Biosystems). The specific primers were as follows: 1) MafA (rat), 5′-AAGGAGGAGGTCATCCGACT-3′ (forward) and 5′-TCTGGAGCTGGCAC TTCTCG-3′ (reverse); 2) MafA (mouse), 5′-AAGCGGCGCACGCTCAAGAA-3′ (forward) and 5′- GGTCCCGCTCCTTGGCCAGA-3′ (reverse); 3) MR, 5′- AGACAATAGTCGGTCTGGGATT-3′ (forward) and 5′- GCTCAGGCTTCCTTGTTGGT-3′ (reverse); and 4) GR, 5′-AATGGGCAAAGGCGATAC-3′ (forward) and 5′-CAGGAGCAAA GCAGAGCAG-3′ (reverse). All data were analyzed using β-actin gene expression as an internal standard.

### RNAi plasmid and recombinant adenovirus construction

GR and MR expression was silenced utilizing specific small interfering RNA (GR-siRNA and MR-siRNA) purchased from Ribobio (Guangzhou, China). The p38 MAPK expression plasmid was constructed by inserting the full-length coding region sequences into pEGFP-N1 vector between PstI and BamHI. The JNK1 expression plasmid was constructed by inserting the full-length coding region sequences into Myc-pCMV5 vector between NdeI and BamHI. The pAdTrack-CMV-MafA expression plasmid was described previously^40^. MafA over-expression adenovirus (Ad-MafA) was constructed and purified as previously described^42^. All constructions used here were sequenced and confirmed to be correct.

### Transient transfection and luciferase reporter assay

MafA transcriptional activity was assessed in Min6 and INS-1 cells using the reporter construct MARE-Luc^43^. We used a plasmid containing the β-galactosidase gene driven by the cytomegalovirus promoter (Clontech Laboratories, Palo Alto, CA, USA) as an internal control. In some experiments, GR-siRNA, pCMV5-JNK1, pcDNA3.0-JNK2, or pEGFP-N1-p38 plasmids were cotransfected into Min6 and INS-1 cells. Twenty-four hours after transfection, the cells were treated with aldosterone for an additional 24 h. The cells were then incubated and harvested for luciferase reporter assays. Luciferase activity was determined as previously described^38^.

### GSIS and KSIS assay

Clonal *β*-cells and isolated primary islets were seeded in a 24-well plate and treated with corresponding drugs as described above for glucose-stimulated insulin secretion (GSIS) and potassium stimulated insulin secretion (KSIS) assays. After incubation for 1 h in glucose-free Krebs-Ringer bicarbonate (KRB) buffer (115 mmol/l NaCl, 4.7 mmol/l KCl, 1.2 mmol/l MgSO4·7H_2_O, 1.2 mmol/l KH_2_PO_4_, 20 mmol/l NaHCO_3_, 16 mmol/l HEPES, 2.56 mmol/l CaCl_2_, 0.2% BSA) and drug solutions, the cells were incubated for 1 h in KRB containing basal glucose (2 mmol/l), stimulatory glucose (20 mmol/l), or KCl (50 mmol/l). The supernatants were obtained for insulin concentration determination using RIA as described^44^.

### TUNEL assay

Min6 cells and INS-1 cells were grown on glass coverslips in the wells of 24-well plates and treated with different concentrates (0 nmol/l, 1 nmol/l, 10 nmol/l, 100 nmol/l, 1000 nmol/l) of aldosterone in serum-free medium for 24, 48 or 72 h. Cells were then fixed and permeabilized, and the TUNEL assay performed according to the manufacturer’s instructions (*In Situ* Cell Death Detection Kit; Roche, Basel, Switzerland). Apoptosis was determined by the TUNEL assay and by scoring cells displaying pycnotic nuclei^40^.

### Statistical analysis

Comparisons were performed using the Student’s t test between two groups or ANOVA in multiple groups. A P value < 0.05 was considered to be statistically significant.

## Additional Information

**How to cite this article**: Chen, F. *et al.* Aldosterone induces clonal *β*-cell failure through glucocorticoid receptor. *Sci. Rep.* **5**, 13215; doi: 10.1038/srep13215 (2015).

## Supplementary Material

Supplementary Information
