# Supplementary material for: Aldosterone induces clonal β-cell failure through glucocorticoid receptor
Source: Sci Rep. 2015 Aug 19;5:13215. doi: 10.1038/srep13215 (PMC4541150; doi:10.1038/srep13215)
Supplement: Supplementary Information [file srep13215-s1.doc]

**Aldosterone induces clonal *β*-cell failure through glucocorticoid receptor**

Fang Chen, Jia Liu, Yanyang Wang, Tijun Wu, Wei Shan, Yunxia Zhu and Xiao Han*

Key Laboratory of Human Functional Genomics of Jiangsu Province, Nanjing Medical University, 140 Hanzhong Road, Nanjing 210029, China.

*Corresponding author: Xiao Han, Ph.D. Mailing address: Key Laboratory of Human Functional Genomics of Jiangsu Province, Nanjing Medical University, 140 Hanzhong Road, Nanjing, 210029, China.

TEL: +86-25-86862731; FAX: +86-25-86862731

E-mail: [hanxiao@njmu.edu.cn](mailto:hanxiao@njmu.edu.cn)

Reprints request should be addressed to Dr. Xiao Han.

**Supplemental Figure-1. Aldosterone induced dysfunction and apoptosis of clonal *β*-cell.** Treatment with aldosterone (10, 100, 1000 nmol/l) for 24 h significantly decreased (A) insulin secretion (white bars, 2 mmol/l glucose; black bars, 50 mmol/l KCl) and (B) insulin content of INS-1 cells. Similar effects on (C) insulin secretion (white bars, 2 mmol/l glucose; gray bars, 20 mmol/l glucose; black bars, 50 mmol/l KCl) and (D) insulin content of rat islets were observed. Min6 cells (E) and INS-1 cells (F) were treated with different concentrations of aldosterone for 24 h, 48 h, and 72 h, and then MTT assays were performed. Aldosterone significantly inhibited viability of Min6 and INS-1 cells. (G) Treatment with aldosterone (10, 100, 1000 nmol/l) for 72 h significantly induced apoptosis of INS-1 cells measured by staining with TUNEL and Hoechst. Apoptosis was determined by scoring the percentage of TUNEL-positive cells. About 2,000 cells were scored for each group in one experiment. *P < 0.05 and **P < 0.01, compared to control.

**Supplemental Figure-2.** Aldosterone induced impairement of INS-1 cells in a GR-dependent manner. After pretreatment with MR antagonist spironolactone (100 nmol/l) or GR antagonist RU486 (1 μmol/l) for 2 h, INS-1 cells were treated with the aldosterone for additional 24 h. The decrease of insulin secretion (A) and insulin content (B) in INS-1 cells induced by aldosterone were significantly reversed by RU486 pretreatment. (C) Transfected with 100 nmol/l GR siRNAs (001, 002, 003) in INS-1 cells for 24 h significantly downregulated GR protein expression. Similar results were obtained by transfected with MR siRNAs (001, 002, 003). After transfected with 100 nmol/l NC, si-GR (003) or si-MR (003) for 24 h, INS-1 cells were treated with aldosterone (100 nmol/l) for 24 h. The decrease of insulin secretion (D) and insulin content (E) in INS-1 cells induced by aldosterone were significantly reversed by transfected with si-GR. (F) Transfected with 100 nmol/l si-GR (003) significantly downregulated GR protein expression in INS-1 cells for different time. (G) INS-1 cells were transfected with 100 nmol/l NC, si-GR (003) or si-MR (003) for 24 h, and then treated with aldosterone (100 nmol/l) for 72 h. The apoptosis of INS-1 cells induced by aldosterone were significantly reversed by transfected with si-GR. **P < 0.01, compared to control; ##*P* < 0.01, compared to NC + aldosterone group.

**Supplemental Figure-3.** Up-regulation of MafA expression protects *β*-cells from aldosterone-induced impairment. (A) Min6 cells, INS-1 cells, mouse islets and rat islets were transfected with MafA over-expression plasmid (P-MafA) or MafA over-expression Adenovirus (Ad-MafA) for 24 h. The overexpression potencies of these plasmid and adenoviruses were confirmed by Western blotting. Transfection with P-MafA in INS-1 cells reversed the aldosterone-induced decrease of insulin secretion (B) and insulin content (C). Infection with Ad-MafA in rat islets reversed the aldosterone-induced decrease of insulin secretion (D) and insulin content(E). (F) Transfection with P-MafA reversed the aldosterone-induced apoptosis of INS-1 cells. **P < 0.01, compared to control. # P < 0.05 and ##P < 0.01, compared to C-plasmid or Ad-GFP combined with aldosterone-treated group.

**Supplemental Figure-4.** The full western blot analysis of MafA expression in Min6, INS-1 and 293A cells was shown. After the treatment of aldosterone for 24h, the expression of MafA protein was determined in Min6, INS-1 and 293A cells. MafA was expressed in Min6 and INS-1 cells, but not 293A cells. Aldosterone treatment significantly inhibited MafA protein expression. Con: control, Ald: aldosterone.


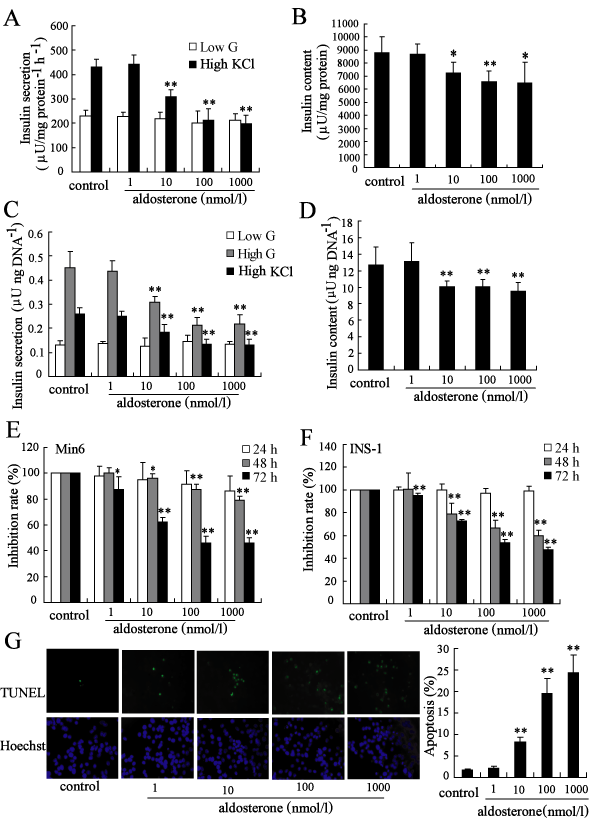


Supplemental Figure-1


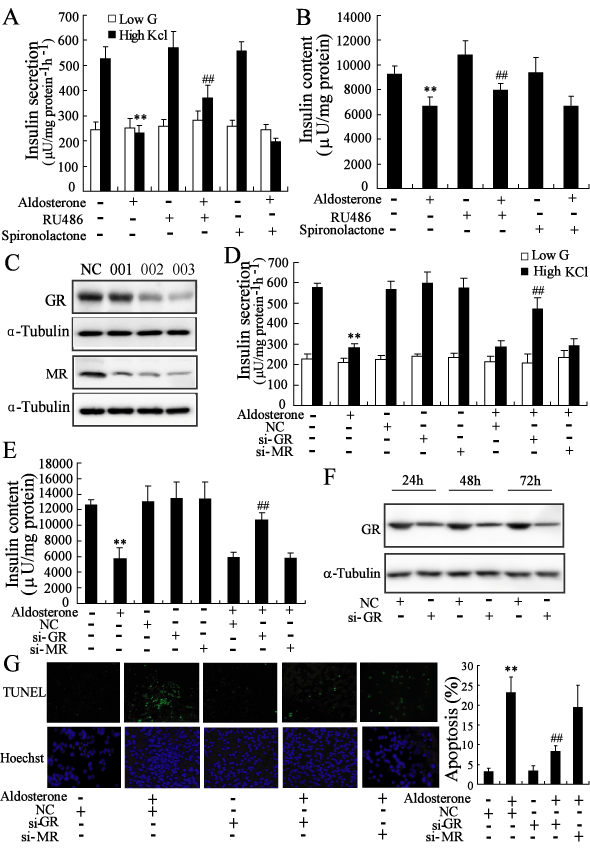


Supplemental Figure-2


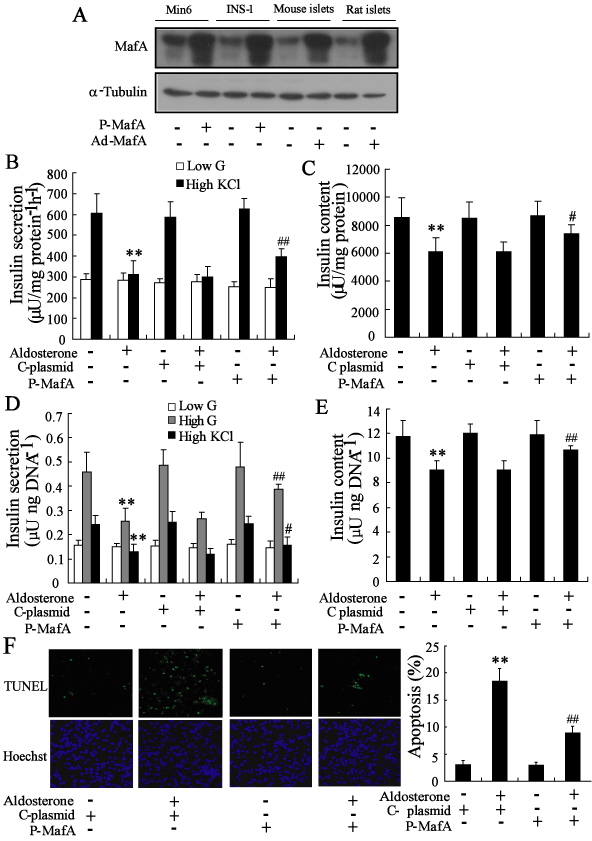


Supplemental Figure-3


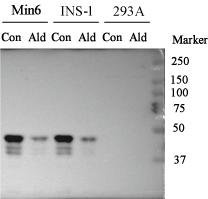


Supplemental Figure-4
